# A CT-based radiomics approach to predict intra-tumoral tertiary lymphoid structures and recurrence of intrahepatic cholangiocarcinoma

**DOI:** 10.1186/s13244-023-01527-1

**Published:** 2023-10-15

**Authors:** Ying Xu, Zhuo Li, Yi Yang, Lu Li, Yanzhao Zhou, Jingzhong Ouyang, Zhen Huang, Sicong Wang, Lizhi Xie, Feng Ye, Jinxue Zhou, Jianming Ying, Hong Zhao, Xinming Zhao

**Affiliations:** 1 Department of Diagnostic Radiology, National Cancer Center/National Clinical Research Center for Cancer/Cancer Hospital, Chinese Academy of Medical Sciences and Peking Union Medical College, Beijing, China; 2 Department of Pathology, National Cancer Center/National Clinical Research Center for Cancer/Cancer Hospital, Chinese Academy of Medical Sciences and Peking Union Medical College, Beijing, China; 3 Department of Hepatobiliary Surgery, National Cancer Center/National Clinical Research Center for Cancer/Cancer Hospital, Chinese Academy of Medical Sciences and Peking Union Medical College, Beijing, China; 4 Key Laboratory of Gene Editing Screening and Research and Development (R&D) of Digestive System Tumor Drugs, Chinese Academy of Medical Sciences and Peking Union Medical College, Beijing, China; 5 Department of Hepatobiliary and Pancreatic Surgery, Affiliated Cancer Hospital of Zhengzhou University, Zhengzhou, Henan China; 6 Magnetic Resonance Imaging Research, General Electric Healthcare, Beijing, China

**Keywords:** Tertiary lymphoid structures, Intrahepatic cholangiocarcinoma, Radiomics, CT, Recurrence

## Abstract

**Purpose:**

To predict the tertiary lymphoid structures (TLSs) status and recurrence-free survival (RFS) of intrahepatic cholangiocarcinoma (ICC) patients using preoperative CT radiomics.

**Patients and methods:**

A total of 116 ICC patients were included (training: 86; external validation: 30). The enhanced CT images were performed for the radiomics model. The logistic regression analysis was applied for the clinical model. The combined model was based on the clinical and radiomics models.

**Results:**

A total of 107 radiomics features were extracted, and after being eliminated and selected, six features were combined to establish a radiomics model for TLSs prediction. Arterial phase diffuse hyperenhancement and AJCC 8th stage were combined to construct a clinical model. The combined (radiomics nomogram) model outperformed both the independent radiomics model and clinical model in the training cohort (AUC, 0.85 vs. 0.82 and 0.75, respectively) and was validated in the external validation cohort (AUC, 0.88 vs. 0.86 and 0.71, respectively). Patients in the rad-score no less than −0.76 (low-risk) group showed significantly better RFS than those in the less than −0.76 (high-risk) group (*p* < 0.001, C-index = 0.678). Patients in the nomogram score no less than −1.16 (low-risk) group showed significantly better RFS than those of the less than −1.16 (high-risk) group (*p* < 0.001, C-index = 0.723).

**Conclusions:**

CT radiomics nomogram could serve as a preoperative biomarker of intra-tumoral TLSs status, better than independent radiomics or clinical models; preoperative CT radiomics nomogram achieved accurate stratification for RFS of ICC patients, better than the postoperative pathologic TLSs status.

**Critical relevance statement:**

The radiomics nomogram showed better performance in predicting TLSs than independent radiomics or clinical models and better prognosis stratification than postoperative pathologic TLSs status in ICC patients, which may facilitate identifying patients benefiting most from surgery and subsequent immunotherapy.

**Key points:**

• The combined (radiomics nomogram) model consisted of the radiomics model and clinical model (arterial phase diffuse hyperenhancement and AJCC 8th stage).

• The radiomics nomogram showed better performance in predicting TLSs than independent radiomics or clinical models in ICC patients.

• Preoperative CT radiomics nomogram achieved more accurate stratification for RFS of ICC patients than the postoperative pathologic TLSs status.

**Graphical Abstract:**

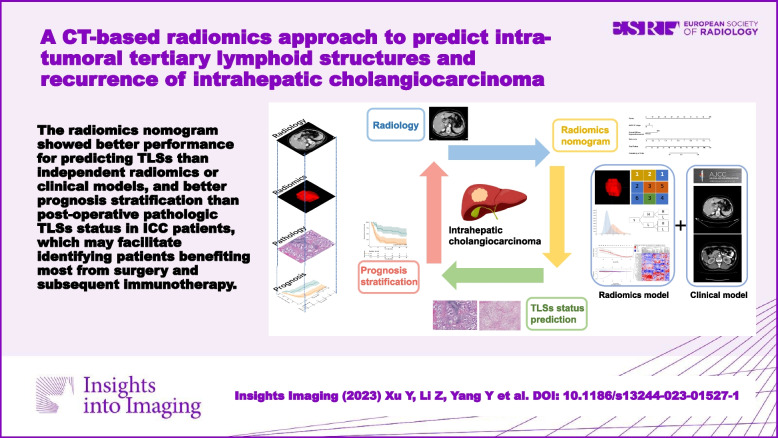

**Supplementary Information:**

The online version contains supplementary material available at 10.1186/s13244-023-01527-1.

## Introduction

Intrahepatic cholangiocarcinoma (ICC) is the second most common primary liver malignancy (10–15%) after hepatocellular carcinoma, with an increasing incidence and mortality globally [1, 2]. Surgical resection is a curative option for ICC. However, most patients (70%) are diagnosed at advanced stages with unresectable tumors due to a lack of specific symptoms, resulting in a dismal prognosis, with a median survival shorter than 12 months [2–4], and high risk of recurrence and metastasis after operation also lead to the poor prognosis [5]. Nowadays, the prognosis prediction of ICC depends on the conventional prognostic factors used in clinical practice such as tumor stage and lymph node status, which is not sufficient for accurate stratification in many cases. Currently, many studies have attempted to find risk factors of prognosis [6–8], but the clinical application value was limited. Accurate identification of recurrence risk factors of ICC is conducive to stratified management and individualized treatment.

The characteristic identification of poor prognosis in cholangiocarcinoma has been transited to the cellular and molecular levels, and it is generally featured by a highly desmoplastic tumor microenvironment (TME) with excessively infiltrating immune and stromal cells [4, 9, 10]. Recently, ectopic aggregates of immune cells with similarities to secondary lymphoid organs (SLO), named tumor-associated tertiary lymphoid structures (TLSs), have attracted extensive attention because of their potential prognostic value and guiding significance of immunotherapy [11]. Ding et al. reported that intra-tumor region TLSs were positively correlated with favorable prognosis whereas peri-tumor region signified worse survival, and performance of the immune classification for ICC was superior to TNM staging system [12]. Zhang et al. demonstrated that the presence of intra-tumoral TLSs was correlated with a good recurrence-free survival (RFS) outcome of perihilar cholangiocarcinoma but not with overall survival (OS) [13].

Considering that TLSs in ICC can only be confirmed through pathological diagnosis, the ability to predict TLSs status preoperatively is of particular importance, especially for unresectable patients. Radiomics extracts, selects, and analyzes the quantitative information that cannot be identified by visual inspection from images to reflect tumoral pathophysiology, intra-tumoral heterogeneity, and cancer phenotype, leading to better clinical decision-making [14, 15]. Computed tomography (CT) plays a vitally important role in non-invasively diagnosing and managing ICC patients [16].

Therefore, the present study aims to preoperatively predict the intra-tumor region TLSs status using the radiomics signature and imaging features on CT images and correlate with survival in ICC patients.

## Patients and methods

### Patient characteristics

**Fig. 1:**
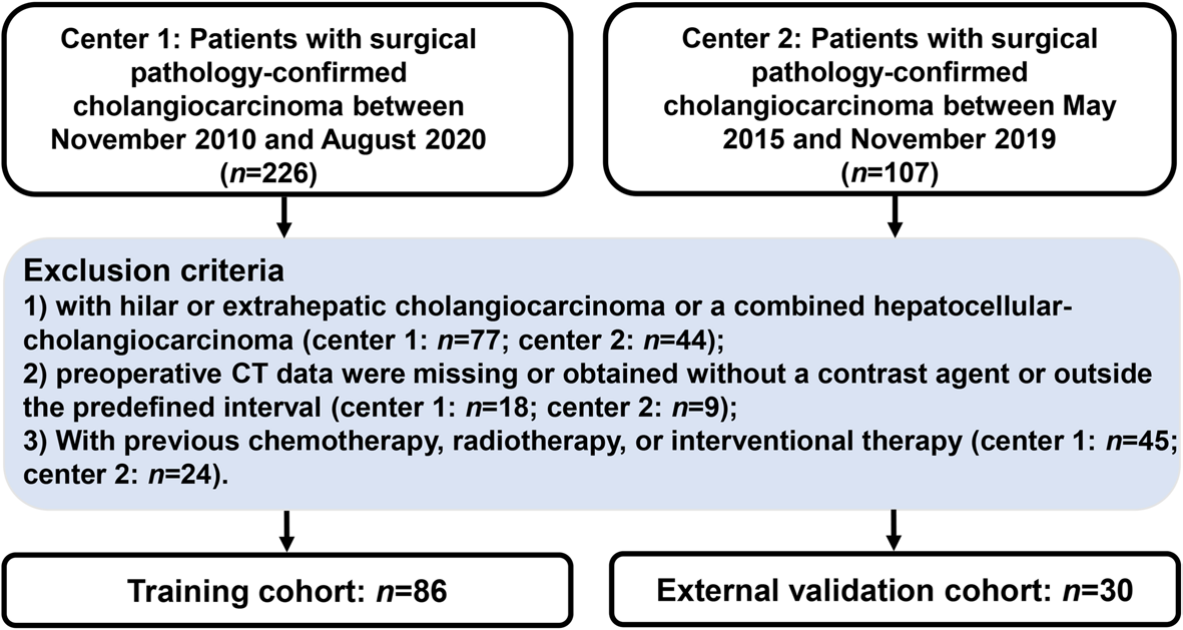
The flowchart of the patient selection process

Preoperative CT scans of 333 patients (training cohort from center 1: 226, external validation cohort from center 2: 107) with surgical pathology-confirmed cholangiocarcinoma were included retrospectively between November 2010 and August 2020, May 2015, and November 2019. The inclusion criteria were as follows: (1) patients with ICC confirmed by histology, (2) patients with preoperative liver dynamic contrast-enhanced (CE) CT data within 1 month before surgery, (3) patients performed with R0 resection (no residual local disease) with postoperative pathological specimens (to identify TLSs), and (4) patients without previous treatment for ICC. The exclusion criteria were as follows: (1) patients with hilar or extrahepatic cholangiocarcinoma or a mixed type of primary liver cancer, (2) preoperative liver CT data were missing or obtained without a contrast agent or outside the predefined interval, and (3) previous treatment for liver lesions (chemotherapy, radiotherapy, or radiofrequency ablation). Based on the inclusion and exclusion criteria, 86 patients with ICC from our institution were included as a training cohort. A flowchart of the patient selection process is shown in Fig. 1. Another validation cohort of 30 patients with ICC was collected from another medical center based on the same criteria as the external validation cohort.

This study was approved by the institutional review board, and the requirement for patient informed consent was waived for this retrospective analysis.

### CT image acquisition

CE-CT imaging of the thorax, abdomen, and pelvis at baseline (within 1 month before surgery) of all patients were performed with a 64-detector row scanner (GE Optima 660 or Discovery 750, General Electric Medical System). Iobitridol injection (320 mg iodine per milliliter, iodixanol injection, Beijing Beilu Pharmaceutical Co., LTD, China) was intravenously injected at a dose of 1.5 ml/kg by using a power injector at a flow rate of 3.0 ml/s. The abdomen CE-CT images on arterial, portal venous, and equilibrium phases were obtained 35, 65, and 150 s after contrast agent administration, respectively (tube voltage, 120 kVp; auto mA settings; pitch, 1.375; rotation time, 0.5 s; thickness, 5 mm).

### Histopathologic analysis

The pathological hematein-eosinsaffron-stained slides of each lesion were reviewed for whole slide images (WSIs) by two pathologists (Z.L. with a 10-year experience and J.M.Y. with a 20-year experience in abdominal pathology). Both pathologists were blinded to the patients’ clinical data and radiological results. Any discrepancy between the two pathologists was discussed to reach a consensus. The existence of intra-tumoral TLSs was assessed morphologically as described previously [12, 17, 18]. Briefly, TLSs were classified into 3 categories according to their maturation stages: (1) lymphoid aggregates (Agg)—vague, ill-defined clusters of lymphocytes; (2) primary lymphoid follicles (Fol-I)—lymphoid follicles without germinal center formation; and (3) secondary lymphoid follicles (Fol-II)—lymphoid follicles with germinal center formation. Intra-tumoral TLSs-negative was defined as tumors without any TLSs, and TLSs-positive was defined as tumors with at least one TLS. Moreover, the following data were systematically recorded: tumor differentiation according to the World Health Organization, nerve invasion, microvascular invasion (MVI), and necrosis. The assessment criteria in the external validation cohort were the same as in the training cohort.

### Clinic-radiologic evaluation

Radiological evaluation was performed by two radiologists (radiologist 1, L.L., with 10 years of experience in abdominal radiology; radiologist 2, Y.X., with 6 years of experience in abdominal radiology) independently on preoperative CT scans. Any discrepancy between the two radiologists was adjudicated by a third senior radiologist (radiologist 3, F.Y., with 20 years of experience in abdominal radiology) to reach a consensus among the three radiologists. All three radiologists were blinded to the patients’ clinical data and pathological results. CT findings of each lesion were evaluated as follows: tumor location, tumor size, satellite nodules, regular morphology, clear border, number, intra-tumoral vessels, macrovascular invasion, portal thrombus, peri-tumoral biliary dilatation, hepatic capsule retraction, and AJCC 8th stage. Moreover, the imaging features of non-enhanced and CE scans were evaluated. The details of the CT imaging findings were presented as Additional file 1: Table S1.

For patients with multiple lesions, the largest lesion was selected for evaluation. Clinical data, including demographic and preoperative tumor markers, were obtained from the medical records.

### Radiomics analysis

Radiomics workflow comprised manual tumor segmentation, feature extraction and selection, volumetric interests (VOIs) fusion, and radiomics model construction. Tumor manual segmentation was performed by radiologist 2 (Y.X., with 5 years of experience in abdominal radiology) under the supervision of senior radiologist 3 (F.Y., with 18 years of experience in abdominal radiology). The VOIs were drawn manually within the visible borders of the tumors while avoiding the blood vessels using the ITK-SNAP v.3.8.0 software (www.itksnap.org) in the axial CE-CT portal vein phase (PVP) imaging. Each VOI consisted of several slices of bi-dimensional regions of interest (ROIs), and the entire volume of each tumor was segmented.

In order to get a standard normal distribution of image densities, the CT scan of each patient was normalized with *Z*-scores according to the following formula to reduce the bias caused by different index dimensions: $f\left(x\right)=\frac{s(x-{\mu }_{x})}{{\sigma }_{x}}$. After adding and averaging the normalized images layer by layer, a total of 107 radiomics features were extracted from each VOI using an in-house software written in Python. The extracted radiomics features included shape (*n* = 14), first-order (*n* = 25), gray-level co-occurrence matrix (GLCM) (*n* = 22), gray-level run length matrix (GLRLM) (*n* = 16), gray-level size zone matrix (GLSZM) (*n* = 16), and gray-level dependence matrix (GLDM) (*n* = 14).

Two feature selection methods, maximum relevance and minimum redundancy (mRMR) and least absolute shrinkage and selection operator (LASSO) [19–23], were used to select the feature for each VOI. First, mRMR was performed to eliminate redundant and irrelevant features. These retained multi-VOI features were finally joined and selected using LASSO to derive the ultimate multi-VOI radiomics model.

A radiomics score (rad-score) was calculated using a linear combination of selected features weighted by their respective LASSO coefficients for each patient, which was the radiomics model. The formula of rad-score was presented as Additional file 1: Supplementary material 2.

### Follow-up

Regular follow-up was conducted every 3 months until the 2nd year after surgery; twice per year in the 3rd, 4th, and 5th years; and once a year after that. Disease recurrence was confirmed by CT, MRI, or positron emission tomography-computed tomography (PET-CT). RFS was defined as the date from the surgery to disease recurrence, or the last follow-up date.

### Statistical analysis

Statistical analyses were performed with SPSS (version 25.0; IBM), R statistical software (version 3.3.3; https://www.r-project.org), and Python (version 3.5.6), PASS 2021, v21.0.3. The chi-square test or Fisher’s exact test was used for categorical variables, and the Mann–Whitney *U* test or Student’s *t*-test was used for continuous variables. The preoperative clinical, radiological variables between TLSs-positive and TLSs-negative in the training cohort were evaluated by univariate analysis, and variables with *p* values of < 0.05 were applied to a multivariate logistic regression analysis. Backward stepwise regression was used to construct the clinical model. The discrimination performance of the clinical, radiomics, and combined models was evaluated using the ROC and the area under the curve (AUC) in the training and external validation cohorts. The corresponding sensitivities, specificities, and accuracies were then compared, and the maximum Youden index of the ROC curve was used to determine the cutoff value for rad-score and nomogram [24]. Calibration curves of the nomogram in the training and external validation cohorts were plotted to assess the consistency between prediction and observation, accompanied by the Hosmer–Lemeshow test. Finally, decision curve analysis (DCA) was conducted to evaluate the clinical usefulness. The survival analyses of the TLSs status, rad-score, and nomogram were prepared using the Kaplan-Meier method with the log-rank test. The performance of TLSs status, rad-score, and nomogram for prognosis prediction was evaluated using the concordance index (C-index). The process of sample size calculation was presented in Additional file 1: Supplementary material 3. *p* < 0.05 was considered statistically significant.

## Results

### Baseline characteristics

A total of 116 (training cohort = 86, external validation cohort = 30) patients were included in the two medical centers. The comparisons of preoperative and postoperative variables between the training and validation cohorts were summarized in Table 1. Patients of age no less than 60 years old were 36 (41.9%) and 10 (33.3%) in training and validation cohorts. All the baseline variables between the two cohorts have no statistical differences.

**Table 1:** The baseline clinic-radiological, pathologic characteristics of ICC patents in the training and validation cohorts

Characteristics	Training cohort (*n* = 86)	Validation cohort (*n* = 30)	*p* value
**Preoperative factors**			
Age ≥ 60, *n* (%)	36 (41.9%)	10 (33.3%)	0.411
Male, *n* (%)	46 (53.5%)	15 (50%)	0.742
HBV-positive, *n* (%)	52 (60.5%)	16 (53.3%)	0.495
Liver cirrhosis, *n* (%)	42 (48.8%)	14 (46.7%)	0.838
Liver steatosis, *n* (%)	23 (26.7%)	7 (23.3%)	0.713
CA199 > 37 U/ml, *n* (%)	38 (44.2%)	14 (46.7%)	0.814
Location, *n* (%)			0.859
Left lobe	50 (58.1%)	18 (60%)	
Right lobe	36 (41.9%)	12 (40%)	
Subcapsular, *n* (%)	62 (72.1%)	21 (70%)	0.827
Satellite nodules, *n* (%)	11 (12.8%)	8 (26.7%)	0.138
Regular morphology, *n* (%)	8 (9.3%)	4 (13.3%)	0.782
Well-defined border, *n* (%)	78 (90.7%)	27 (90%)	1.000
Number, *n* (%)			0.231
1	80 (93%)	25 (83.3%)	
> 1	6 (7%)	5 (16.7%)	
Diameter > 5 cm, *n* (%)	46 (53.5%)	16 (53.3%)	0.988
Macrovascular invasion, *n* (%)	73 (84.9%)	24 (80%)	0.737
Suspicious lymph node metastasis, *n* (%)	21 (24.4%)	6 (20%)	0.622
AJCC 8th stage, *n* (%)			0.288
1	47 (54.7%)	16 (53.3%)	
2	13 (15.1%)	8 (26.7%)	
3	26 (30.2%)	6 (20%)	
CT non-enhanced scan density, *n* (%)			0.255
Homogeneous low	15 (17.4%)	2 (6.7%)	
Inhomogeneous low	71 (82.6%)	28 (93.3%)	
Arterial diffuse hyperenhancement, *n* (%)	18 (20.9%)	5 (16.7%)	0.614
Arterial peripheral rim enhancement, *n* (%)	46 (53.5%)	18 (60%)	0.537
Arterial diffuse hypoenhancement, *n* (%)	22 (25.6%)	7 (23.3%)	0.807
Centripetal enhancement, *n* (%)	40 (46.5%)	14 (46.7%)	0.988
Wash in and wash out, *n* (%)	13 (15.1%)	5 (16.7%)	1.000
Persistent enhancement, *n* (%)	19 (22.1%)	8 (26.7%)	0.610
Peritumoral arterial enhancement, *n* (%)	25 (29.1%)	11 (36.7%)	0.439
Intra-tumoral vessels, *n* (%)	13 (15.1%)	3 (10%)	0.695
Portal thrombus, *n* (%)	10 (11.6%)	3 (10%)	1.000
Biliary dilatation, *n* (%)	21 (24.4%)	7 (23.3%)	0.905
Hepatic capsule retraction, *n* (%)	61 (70.9%)	21 (70%)	0.923
**Postoperative factors**			
TLSs-positive, *n* (%)	27 (31.4%)	10 (33.3%)	0.845
Differentiation, *n* (%)			0.951
Poor	55 (64%)	19 (63.3%)	
Well-moderate	31 (36%)	11 (36.7%)	
Nerve invasion, *n* (%)	25 (29.1%)	5 (16.7%)	0.182
MVI-positive, *n* (%)	29 (33.7%)	9 (30%)	0.708
Necrosis, *n* (%)	76 (88.4%)	28 (93.3%)	0.674
Type of hepatic resection, *n* (%)			0.699
Major	31 (36%)	12 (40%)	
Minor	55 (64%)	18 (60%)	
Adjuvant therapy, *n* (%)	29 (33.7%)	8 (26.7%)	0.475

*HBV* hepatitis B virus, *CA199* carbohydrate antigen 199, *AJCC* American Joint Committee on Cancer, *TLSs* tertiary lymphoid structures, *MVI* microvascular invasion

### Clinical model construction

The preoperative clinical and radiological variables of TLSs-positive and TLSs-negative groups in the training cohorts are summarized in Table 2. Univariate analysis indicated that the arterial diffuse hyperenhancement, arterial peripheral rim enhancement, AJCC 8th stage, and diameter were significantly different between the TLSs-positive and TLSs-negative groups in the training cohort (*p* < 0.001; 0.003; 0.014; 0.039, respectively). Multivariate logistic regression analysis showed that the arterial phase diffuse hyperenhancement and AJCC 8th stage were combined to construct a clinical model, and the arterial phase diffuse hyperenhancement was an independent factor for differentiating TLSs status (*p* = 0.035).

**Table 2:** Univariate and multivariate analyses of the preoperative clinical, radiologic variables between the TLSs-positive and TLSs-negative groups in the training cohort

Characteristics	Univariate analysis			Multivariate analysis	
	TLSs-positive (*n* = 27)	TLSs-negative (*n* = 59)	*p* value	OR (95% CI)	*p* value
Age ≥ 60, *n* (%)	12 (44.4%)	24 (40.7%)	0.742		
Male, *n* (%)	12 (44.4%)	34 (57.6%)	0.255		
HBV-positive, *n* (%)	17 (63%)	35 (59.3%)	0.749		
Liver cirrhosis, *n* (%)	14 (51.9%)	28 (47.5%)	0.705		
Liver steatosis, *n* (%)	6 (22.2%)	17 (28.8%)	0.522		
CA199 > 37 U/ml, *n* (%)	11 (40.7%)	27 (45.8%)	0.663		
Location, *n* (%)			0.887		
Left lobe	16 (59.3%)	34 (57.6%)			
Right lobe	11 (40.7%)	25 (42.4%)			
Subcapsular, *n* (%)	17 (63%)	45 (76.3%)	0.202		
Satellite nodules, *n* (%)	1 (3.7%)	10 (16.9%)	0.174		
Regular morphology, *n* (%)	4 (14.8%)	4 (6.8%)	0.429		
Well-defined border, *n* (%)	25 (92.6%)	53 (89.8%)	0.993		
Number, *n* (%)			0.726		
1	26 (96.3%)	54 (91.5%)			
> 1	1 (3.7%)	5 (8.5%)			
Diameter > 5 cm, *n* (%)	10 (37%)	36 (61%)	0.039*		0.365
Macrovascular invasion, *n* (%)	3 (11.1%)	10 (16.9%)	0.706		
Lymph node metastasis, *n* (%)	4 (14.8%)	17 (28.8%)	0.161		
AJCC 8th stage, *n* (%)			0.014*	0.598 (0.309–1.155)	0.126
1	21 (77.8%)	26 (44.1%)			
2	2 (7.4%)	11 (18.6%)			
3	4 (14.8%)	22 (37.3%)			
CT non-enhanced scan density, *n* (%)			0.628		
Homogeneous low	6 (22.2%)	9 (15.3%)			
Inhomogeneous low	21 (77.8%)	50 (84.7%)			
Arterial diffuse hyperenhancement, *n* (%)	13 (48.1%)	5 (8.5%)	< 0.001*	4.801 (1.116–20.649)	0.035*
Arterial peripheral rim enhancement, *n* (%)	8 (29.6%)	38 (64.4%)	0.003*		0.473
Arterial diffuse hypoenhancement, *n* (%)	6 (22.2%)	16 (27.1%)	0.629		
Centripetal enhancement, *n* (%)	12 (44.4%)	28 (47.5%)	0.795		
Wash in and wash out, *n* (%)	5 (18.5%)	8 (13.6%)	0.786		
Persistent enhancement, *n* (%)	6 (22.2%)	13 (22%)	0.984		
Peritumoral arterial enhancement, *n* (%)	8 (29.6%)	17 (28.8%)	0.938		
Intra-tumoral vessels, *n* (%)	5 (18.5%)	8 (13.6%)	0.786		
Portal thrombus, *n* (%)	4 (14.8%)	6 (10.2%)	0.794		
Biliary dilatation, *n* (%)	8 (29.6%)	13 (22%)	0.447		
Hepatic capsule retraction, *n* (%)	17 (63%)	44 (74.6%)	0.271		

*TLSs* tertiary lymphoid structures, *HBV* hepatitis B virus, *CA199* carbohydrate antigen 199, *AJCC* American Joint Committee on Cancer

^*^Statistically significant

### Feature selection and radiomics model construction

**Fig. 2:**
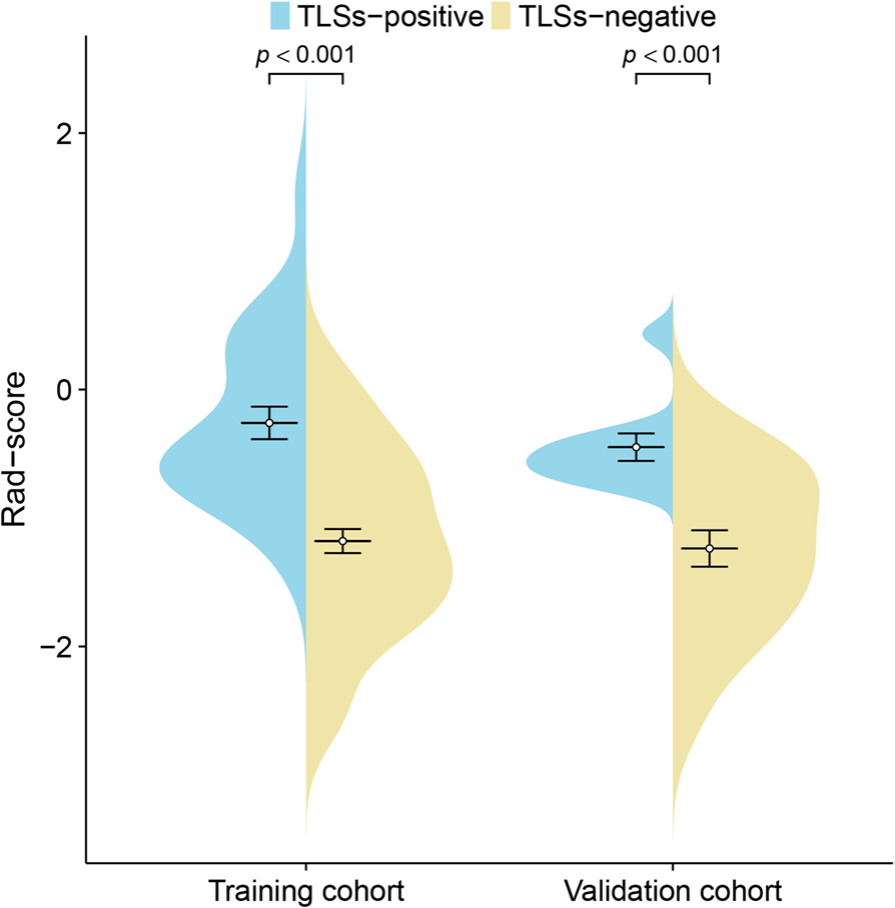
Violin plots comparing the rad-score of the TLSs-positive and TLSs-negative groups in the training and external validation cohorts

A total of 107 features were extracted, and 30 features were retained after eliminating redundant and irrelevant features using mRMR. LASSO regression analysis was used to select 6 features to derive a radiomics model. We compared the rad-scores of TLSs-positive and TLSs-negative groups in the training and external validation cohorts, respectively. Patients in the TLSs-positive group showed a significantly higher rad-score than the TLSs-negative group in the two cohorts (−0.26 ± 0.66 vs. −1.18 ± 0.72, *p* < 0.001; −0.45 ± 0.34 vs. −1.24 ± 0.63, *p* < 0.001) (Fig. 2).

### Combined model construction and performance evaluation of three models

**Fig. 3:**
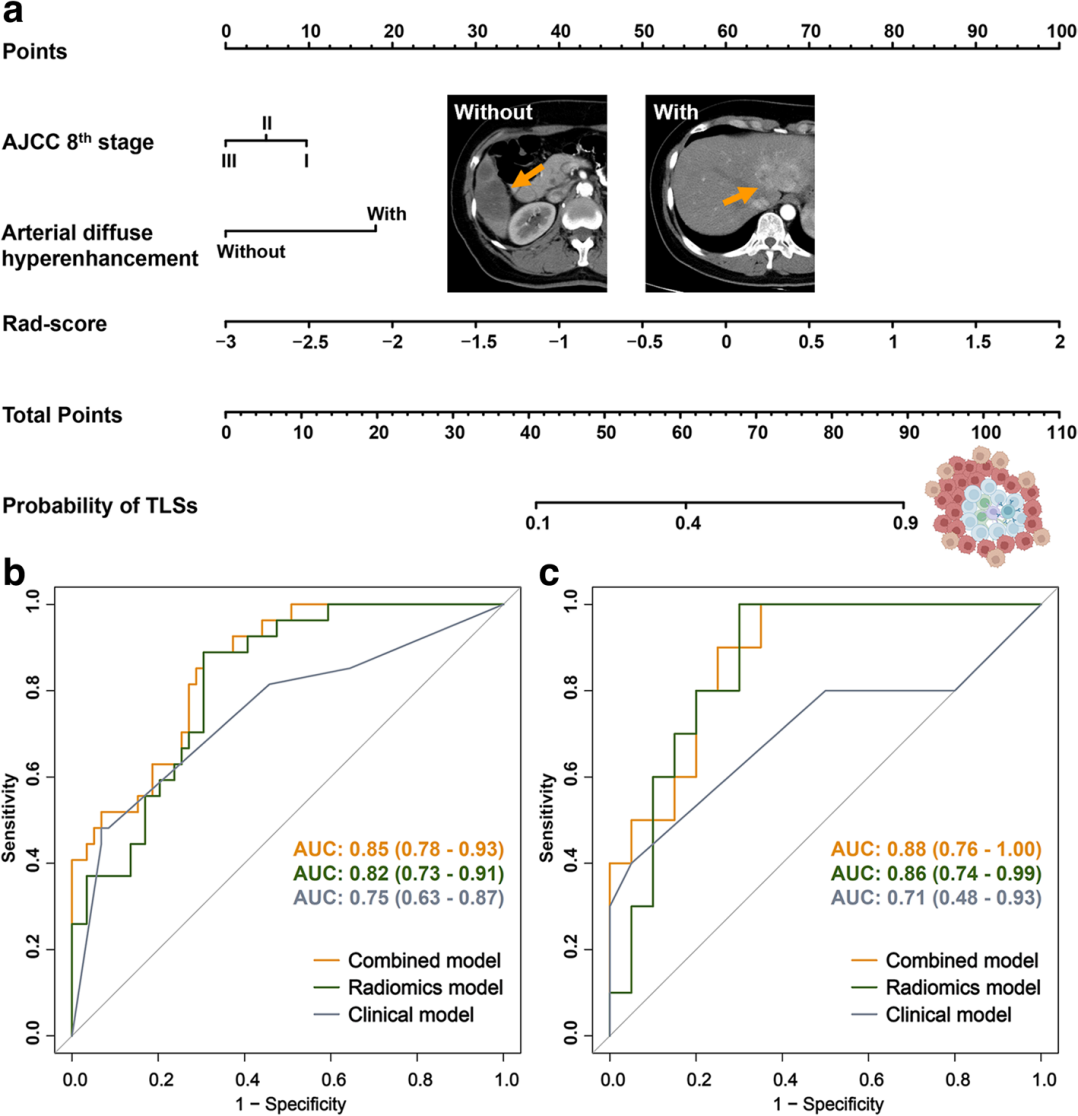
The radiomics nomogram model integrating clinical model (AJCC 8th stage, arterial diffuse hyperenhancement) and radiomics model (rad-score) (**a**). ROC curves of radiomics model, clinical model, and combined model were compared in the training (**b**) and external validation (**c**) cohorts

The final radiomics nomogram model integrated the clinical model and radiomics model (Fig. 3a). The combined (radiomics nomogram) model outperformed both the independent radiomics model and clinical model in the training cohort (AUC, 0.85 vs. 0.82 and 0.75, respectively) and was validated in the external validation cohort (AUC, 0.88 vs. 0.86 and 0.71, respectively) (Table 3, Fig. 3b, c).

**Table 3:** Performance comparison of the clinical model, radiomics signature, and radiomics nomogram in the training and external validation cohorts

Model	Training cohort (*n* = 86)	External validation cohort (*n* = 30)
**Clinical model**		
Sensitivity	0.76	0.80
Specificity	0.80	0.76
Accuracy	0.79	0.77
AUC (95% CI)	0.75 (0.63–0.87)	0.71 (0.48–0.93)
**Radiomics model**		
Sensitivity	0.89	1.0
Specificity	0.69	0.70
Accuracy	0.76	0.80
AUC (95% CI)	0.82 (0.73–0.91)	0.86 (0.74–0.99)
**Radiomics nomogram**		
Sensitivity	0.57	0.60
Specificity	0.93	0.93
Accuracy	0.76	0.77
AUC (95% CI)	0.85 (0.78–0.93)	0.88 (0.76–1.00)

Favorable calibrations of the nomogram were obtained in the training and external validation cohorts (Additional file 1: Fig. S1A and B). The Hosmer–Lemeshow test yielded *p* values of 0.19 and 0.26. DCA curves of the three models were presented in Additional file 1: Fig. S1C. The rad-score and nomogram score of each patient in the training cohort were presented in Additional file 1: Fig. S2.

### Correlations of TLSs status, rad-score, and nomogram with recurrence

**Fig. 4:**
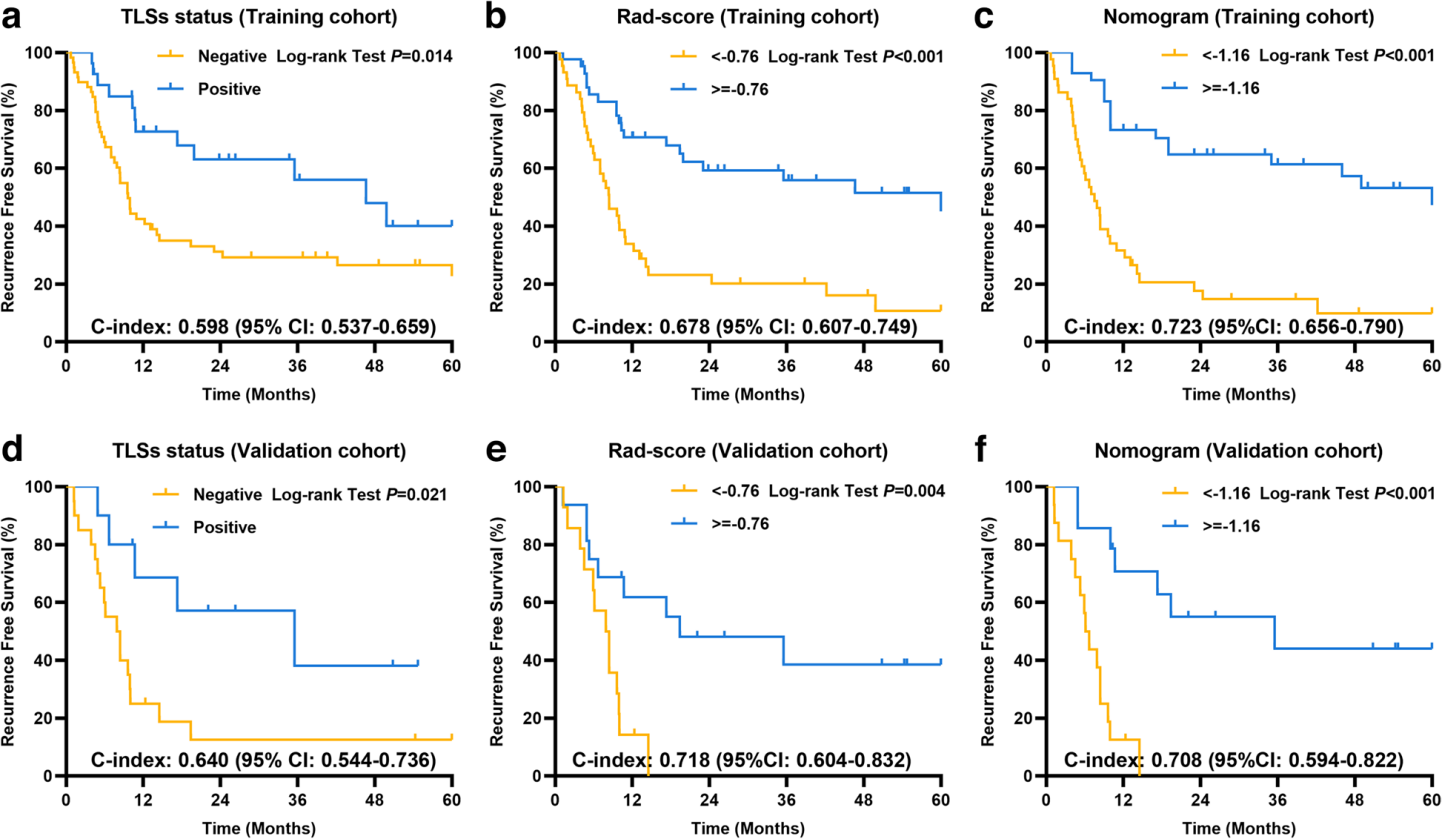
Kaplan-Meier curves for RFS of patients with ICC as categorized by the TLSs status, rad-score (cutoff value = −0.76), and nomogram score (cutoff value = −1.16) in the training (**a**–**c**) and external validation cohorts (**d**–**f**)

For the 86 ICC patients in the training cohort, fifty-four patients (62.8%) experienced recurrence during a median follow-up duration of 50.8 months (95% confidence intervals [CIs]: 29.5–72.0 months). The median RFS was 13.1 months (95% CIs, 5.0–21.2 months). Patients of intra-tumoral TLSs-positive showed significantly better RFS than those of TLSs-negative (median RFS: 46.6; 95% CIs, 25.6–67.7 months vs. median RFS: 9.6; 95% CIs, 7.9–11.3 months, C-index = 0.598 [95% CI, 0.537–0.659], *p* = 0.014; Fig. 4a). The 6-, 12-, 24-, 36-, 48-, and 60-month cumulative RFS rates of the TLSs-positive group were 88.7%, 72.8%, 63.1%, 56.0%, 48.0%, and 40.0%, and 69.1%, 42.5%, 31.1%, 29.2%, 26.5%, and 22.7% for the TLSs-negative group, respectively.

The radiomics model divides ICC patients into a high-risk group with a cutoff value of less than −0.76 and vice versa into a low-risk group (Fig. 4b; *p* < 0.001, C-index = 0.678 [95% CI, 0.607–0.749]). The median RFS of the high- and low-risk groups was 8.4 months (95% CIs, 7.0–12.2 months) and 60.0 months (95% CIs, 19.9 months—not reached), respectively. The 6-, 12-, 24-, 36-, 48-, and 60-month cumulative RFS rates of the low-risk group were 85.5%, 70.7%, 59.4%, 55.9%, 51.6%, and 45.1%, and 65.3%, 33.9%, 23.1%, 20.2%, 16.2%, and 10.8% for the high-risk group, respectively.

The nomogram model divides ICC patients into a high-risk group with a cutoff value of less than −1.16 and vice versa into a low-risk group (Fig. 4c; *p* < 0.001, C-index = 0.723 [95% CI, 0.656–0.790]). The median RFS of the high- and low-risk groups was 7.5 months (95% CIs, 5.5–10.9 months) and 60.0 months (95% CIs, 35.5 months—not reached), respectively. The 6-, 12-, 24-, 36-, 48-, and 60-month cumulative RFS rates of the low-risk group were 92.7%, 72.9%, 64.5%, 61.1%, 57.1%, and 47.1%, and 58.3%, 31.6%, 17.7%, 14.8%, 9.8%, and 9.8% for the high-risk group, respectively.

**Fig. 5:**
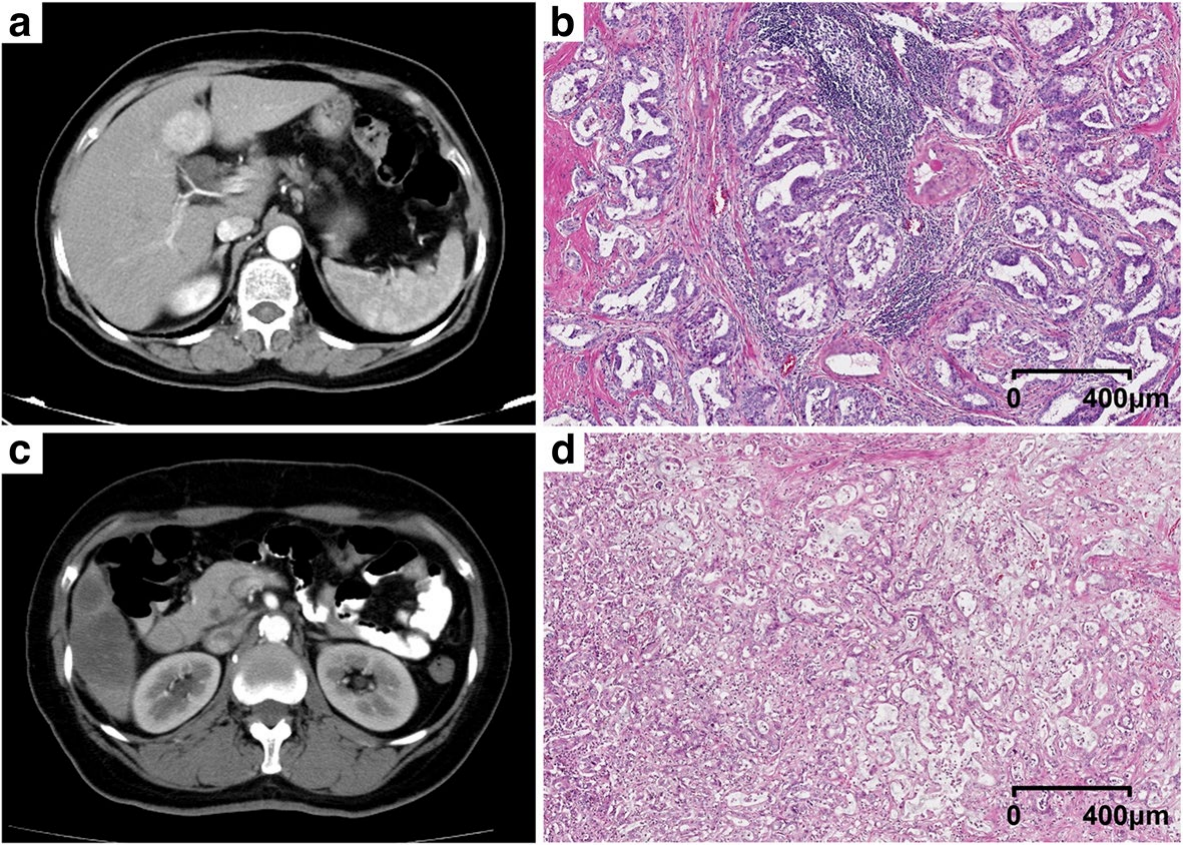
Two representative cases with TLSs-positive (**a**, **b**) and TLSs-negative (**c**, **d**) tumors were presented. For the TLSs-positive tumor, the arterial phase of enhanced CT showed diffuse hyperenhancement (**a**); the rad-score was 0.43; the nomogram score was 2.83; the pathological hematein-eosin saffron-stained slide (**b**) showed the immune infiltration in TLSs; the RFS of the patient was 25.30 months. For the TLSs-negative tumor, the arterial phase of enhanced CT showed diffuse hypoenhancement (**c**); the rad-score was −0.78; the nomogram score was −1.63; the pathological hematein-eosin saffron-stained slide (**d**) showed diffuse tumor cells and no TLSs in the tumor; the RFS of the patient was 1.20 months

For the 30 ICC patients in the external validation cohort, the median RFS was 9.6 months (95% CIs, 7.4–11.8). Patients of intra-tumoral TLSs-positive showed significantly better RFS than those of TLSs-negative (median RFS: 35.5; 95% CIs, 10.7–71.2 months vs. median RFS: 7.9; 95% CIs, 2.9–12.9 months, *p* = 0.021, C-index = 0.640 [95% CI, 0.544–0.736]; Fig. 4d). Patients of the rad-score no less than −0.76 (low-risk) group showed significantly better RFS than those of the rad-score less than −0.76 (high-risk) group (median RFS: 19.4; 95% CIs, 6.7–47.2 months vs. median RFS: 7.9; 95% CIs, 3.7–12.1 months, *p* = 0.004, C-index = 0.718 [95% CI, 0.604–0.832]; Fig. 4e). Patients of the nomogram score no less than −1.16 (low-risk) group showed significantly better RFS than those of the nomogram score less than −1.16 (high-risk) group (median RFS: 35.5; 95% CIs, 17.3–77.9 months vs. median RFS: 6.1; 95% CIs, 4.5–7.7 months, *p* < 0.001, C-index = 0.708 [95% CI, 0.594–0.822]; Fig. 4f). Discrimination of radiological and pathologic images between TLSs-positive and TLSs-negative tumors was shown as Fig. 5.

## Discussion

In this study, we demonstrated that the presence of intra-tumoral TLSs was an effective predictor of favorable prognosis for ICC, which is consistent with the previous studies [4, 12]. Previous studies also indicated that intra-tumoral TLSs predicted a better response to immunotherapy independent of PD-L1 expression status and CD8+ T cell density, not only for ICC, but also for other types of solid tumors including hepatocellular carcinoma and melanoma [4, 25–28]. As a result, we attempted to preoperatively predict TLSs status non-invasively. Clinical and radiomics models were constructed independently with AUCs of 0.75 and 0.82, and the prediction performance of the combined model integrating clinical and radiomics models proved to be the best among the three models, with an AUC of 0.85. Moreover, the rad-score and combined model divided ICC patients into a high- and low-risk group with a cutoff value of −0.76 and −1.16, respectively, which achieved more accurate RFS stratification than the postoperative TLSs status (*p* < 0.001; *p* < 0.001; *p* = 0.014).

In spite of the significance of intra-tumoral TLSs status, no studies have investigated the correlations between imaging features and TLSs status. Min et al. found that the risk of death and recurrence in patients with ICC with arterial diffuse hyperenhancement on MRI images were lower than those with diffuse hypoenhancement or peripheral rim enhancement [29]. Our study demonstrated that ICC of TLSs-positive had more frequent arterial diffuse hyperenhancement than those of TLSs-negative. However, a clear illustration regarding arterial hyperenhancement was not proposed but was presumed to be related to more cellular areas with less fibrosis [29, 30]. But what is the cellular component that causes the high arterial hyperenhancement is unclear. The composition of TLSs included CD20^+^ B cells, CD3^+^ T cells, CD4^+^ T follicular helper (TFH) cells, CD8^+^ cytotoxic T cells, CD4^+^ T helper 1 (TH1) cells, regulatory T cells (Tregs), and CD21^+^ follicular dendritic cells (FDCs) [31–34], which is defined immune infiltrates in tumors. We assumed that arterial diffuse hyperenhancement could be explained by the immune infiltration and high endothelial venules (HEVs) [35], which might be associated with a preferable prognosis. Our study also demonstrated that the ICC of TLSs-positive had more frequent stage I of AJCC 8th stage. There are no previous studies investigating the preoperative predictors of TLSs in patients with ICC. One study demonstrated that TNM stage was significantly different between the TLSs-positive and TLSs-negative groups for perihilar cholangiocarcinoma, and a higher proportion of TLSs-positive patients were evaluated as TNM stages 1 and 2 than the TLSs-negative patients [13]. This is consistent with our findings. We presumed that the TLSs-positive might be associated with more benign biological behavior and lower invasive, metastatic potential, which might lead to a lower TNM stage.

A total of 6 radiomics signatures were selected to derive a radiomics model, including three first-order, two GLRLM, and one GLDM features. We assumed that the difference in intra-tumoral heterogeneity between the TLSs-positive and TLSs-negative tumors may be associated with different radiomics signatures. Radiomics was reported to have great potential for extracting the biological characteristics and prognostic information of ICC [8, 36–42]. Chu et al. presented a CT-based radiomics model for the prediction of futile resection before surgery in ICC patients, with better performance than clinical information [40]. Ji et al. developed a radiomics model for the prediction of lymph node metastasis (AUC = 0.85) [41]. One shape feature, two first-order features, one GLDM feature, one GLCM feature, one GLRLM feature, and two GLSZM features were selected and constructed in the radiomics model. Song et al. developed a combined radiomics model to preoperatively predict the early recurrence of ICC patients [42]. The combined clinical-radiomics model included 15 radiomics features and 3 clinical features (CA19-9 > 1000 U/ml, vascular invasion, and tumor margin), resulting in the AUCs of 0.974 in the derivation cohort, which are higher than the AJCC 8th TNM staging system. Zhang et al. developed a radiomics model based on MR images for preoperative evaluation immunophenotyping and survival in ICC patients. Four texture features were selected, and three of them were wavelet features. Each feature had the favorable ability to discriminate the immunophenotyping, and the best performance was achieved by a combination of all features, with an AUC of 0.919 [36]. Zhang et al. found that three wavelet features in the arterial phase (AP), three wavelet features in the portal vein phase (PVP), and one first-order features in PVP could preoperatively predict PD-1/PD-L1 expression for ICC [38]. All the studies indicated the potential predictive ability of radiomics in tumor heterogeneity, immunophenotyping, and microscopic pathological features.

Based on the Radiomics Quality Score (RQS) developed by Lambin et al., our study got a score of 16 [43]. According to a systematic review about cholangiocarcinoma, the highest RQS was 18, and there were only 7 studies (18.4%) with ≥ 15 scores. Thirty (79.0%) studies were performed at one institution, and all the included studies were conducted retrospectively [44]. For our study, patients from two centers were included retrospectively. Moreover, the most common study aims included differential diagnosis against other hepatic lesions, prediction of survival after surgical resection, prediction of lymph node metastases, and prediction of therapeutic response to radioembolization [44]. Currently, none of the studies covered the prediction of TLSs. Consequently, this study containing external validation cohort explored an innovative topic, with fairly high quality.

There are some limitations in this study. First, this is a retrospective study, and selection bias is inevitable. For example, patients without preoperative CT data or obtained without a contrast agent or outside the predefined interval were excluded. Second, the sample size was limited, and studies in a larger cohort of ICC patients were further needed. Third, the volatility of scores caused by nomograms is inevitable, and we are trying web applications for the construction of dynamic prediction tools [45]. Fourth, the extraction of radiomics features was only from the portal vein phase, not the arterial phase, and we will attempt to perform feature extraction from the arterial phase in CT and from various phases in MRI next step.

In conclusion, this study demonstrated that CT radiomics nomogram could serve as a preoperatively predictive biomarker of intra-tumoral TLSs status, better than independent radiomics or clinical models; preoperative CT radiomics nomogram achieved more accurate stratification for RFS of ICC patients, better than the postoperative pathologic TLSs status.

### Supplementary Information

**Additional file 1: Supplementary material 1. Table S1.** The CT findings and density characteristics. **Supplementary material 2.** The formula of the rad-score. **Supplementary material 3.** The sample size calculation. **Supplementary material 4. Fig. S1.** Calibrations of the nomogram in the training (A), external validation (B) cohorts. Decision curve analysis for three models (C). The y-axis indicates the net benefit and x-axis indicates threshold probability. The yellow line, grey line, and green line represent net benefit of the radiomics nomogram, the radiomics model and the clinical model, respectively. The radiomics nomogram model had the highest net benefit compared with the other two models. **Supplementary material 5. Fig. S2.** The waterfall plot of the rad-score and nomogram points of each patient in training cohort.

## Data Availability

The datasets used or analyzed during the current study are available from the corresponding author upon reasonable request. Requests to access these datasets could be directed to dr_fengye_ncc@163.com.

## Abbreviations

Agg: Lymphoid aggregates
AUC: Area under the curve
CE: Contrast-enhanced
CIs: Confidence intervals
CT: Computed tomography
DCA: Decision curve analysis
Fol-I: Primary lymphoid follicles
Fol-II: Lymphoid follicles with germinal center formation
GLCM: Gray-level co-occurrence matrix
GLDM: Gray-level dependence matrix
GLRLM: Gray-level run length matrix
GLSZM: Gray-level size zone matrix
ICC: Intrahepatic cholangiocarcinoma
LASSO: Least absolute shrinkage and selection operator
MRI: Magnetic resonance imaging
mRMR: Maximum relevance and minimum redundancy
OS: Overall survival
PVP: Portal vein phase
rad-score: Radiomics score
RFS: Recurrence-free survival
ROIs: Regions of interest
SD: Standard deviation
SLO: Secondary lymphoid organs
TLSs: Tertiary lymphoid structures
TME: Tumor microenvironment
VOIs: Volumetric interests
WSIs: Whole slide images
