# Supplementary material for: A CT-based radiomics approach to predict intra-tumoral tertiary lymphoid structures and recurrence of intrahepatic cholangiocarcinoma
Source: Insights Imaging. 2023 Oct 15;14:173. doi: 10.1186/s13244-023-01527-1 (PMC10577112; doi:10.1186/s13244-023-01527-1)
Supplement: Supplementary file 1 — Additional file 1: Supplementary material 1. Table S1. The CT findings and density characteristics. Supplementary material 2. The formula of the rad-score. Supplementary material 3. The sample size calculation. Supplementary material 4. Fig. S1. Calibrations of the nomogram in the training (A), external validation (B) cohorts. Decision curve analysis for three models (C). The y-axis indicates the net benefit and x-axis indicates threshold probability. The yellow line, grey line, and green line represent net benefit of the radiomics nomogram, the radiomics model and the clinical model, respectively. The radiomics nomogram model had the highest net benefit compared with the other two models. Supplementary material 5. Fig. S2. The waterfall plot of the rad-score and nomogram points of each patient in training cohort. [file 13244_2023_1527_MOESM1_ESM.docx]

**A CT-based radiomics approach to predict intra-tumoral tertiary lymphoid structures and recurrence of intrahepatic cholangiocarcinoma**

**ELECTRONIC SUPPLEMENTARY MATERIAL**

**Supplementary material 1: Table 1.** The CT findings and density characteristics.

| CT findings and density characteristics | Definition[1-6] |
| --- | --- |
| Non-enhanced scan |  |
| hetegeneous low density | Hetegeneous lower density than liver parenchyma on non-enhanced scan. |
| homogeneous low density | Homogeneous lower density than liver parenchyma on non-enhanced scan. |
| Arterial phase |  |
| Diffuse hyperenhancement | Homogeneous higher density than liver parenchyma in >70% of the tumor area on arterial phase enhancement. |
| Peripheral rim hyperenhancement | Peripheral ring-like hyper-density in 10%-70% tumor area with central hypodensity areas on arterial phase enhancement. |
| Diffuse hypoenhancement | Homogeneous lower density than liver parenchyma on arterial phase enhancement. |
| Peritumoral enhancement | Gross hyperenhancement outside the tumor border in the arterial phase, becoming iso-density in later dynamic phases compared with the background liver parenchyma. |
| Enhancement pattern |  |
| Wash in and wash out | Arterial hyperenhancement with iso-density or hypodensity in the portal venous and delayed phase. |
| Centripetal enhancement | Rim or peripheral arterial-phase enhancement, and centripetal enhancement on the delayed phase. |
| Persistent enhancement | Hyperenhancement on the arterial phase and persistent enhancement in the portal venous and delayed phase. |
| Tumor location |  |
| Left or right lobe | Left lobe consists of 2-4 segments of liver and right lobe consists of 5-8 segments. |
| Subcapsular | The lesion is adjacent to the liver capsule on the images. |
| Tumor size | Measurement of the maximum diameter in the delayed phase on axial CT scans. |
| Satellite nodules | Distinct tumor nodules in the vicinity of the main tumor. |
| Regular morphology | Tumor without a budding portion protruding into the liver parenchyma. |
| Clear border | Tumor had smooth margins and the boundary with the surrounding liver parenchyma was clear at each phase. |
| Intratumor vessels | Blood vessels including hepatic arteries, hepatic veins, portal veins, and their branches go through the tumor. |
| Macrovascular invasion | A mass invasion into the main portal veins and hepatic vein or their branches, or the inferior vena cava. |
| Hepatic capsule retraction | Invagination or focal flattening of the typical smooth contour of the liver capsule. |

**Supplementary material 2:**

*Rad-score = --0.719*original_glrlm_RunEntropy+-0.057*original_gldm_LargeDependenceLowGrayLevelEmphasis+-0.053*original_firstorder_Skewness+0.007*original_firstorder_Kurtosis+0.087*original_firstorder_lz10Percentile+0.26*original_glrlm_HighGrayLevelRunEmphasis + -0.89*

**Supplementary material 3:**

The sample size calculation. The sample size was calculated with the PASS software 2021, v21.0.3. The median survival time was 46.6 and 9.6 months in the treatment (TLSs-positive) and control (TLSs-negative) groups which based on the training cohort data, with alpha of 0.05 and beta of 0.1952. The power was 0.80 and alternative hypothesis was one-sided. Based on the above real-world data in the training cohort, the ratio of positive to negative group was 1:2, and we set the positive: negative group as 1:2. Finally the sample size should be 10 in the TLSs-positive group and 20 in the TLSs-negative. And this is the reason why we included the 30 patients as the external validation cohort. The calculation result is as follows:


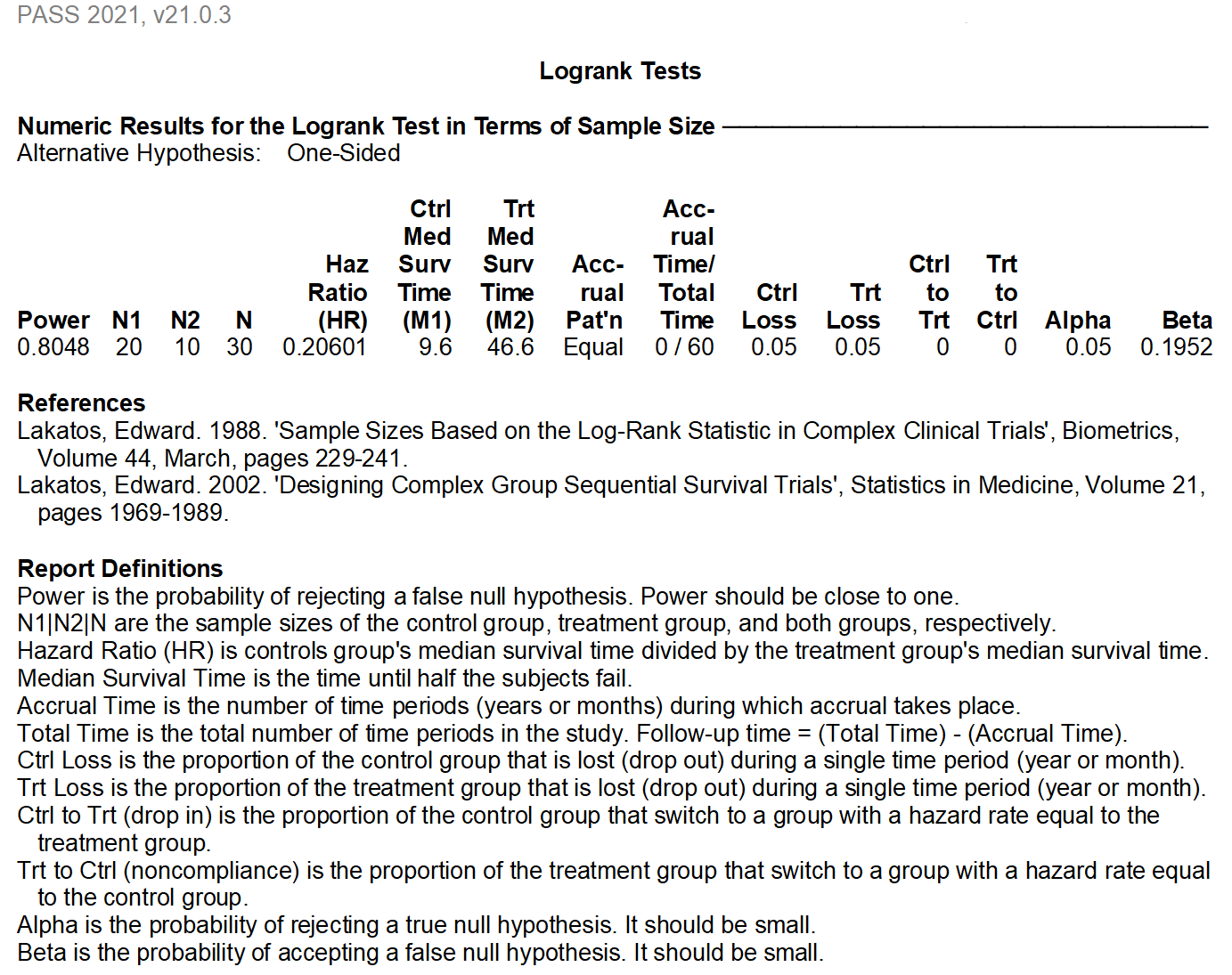


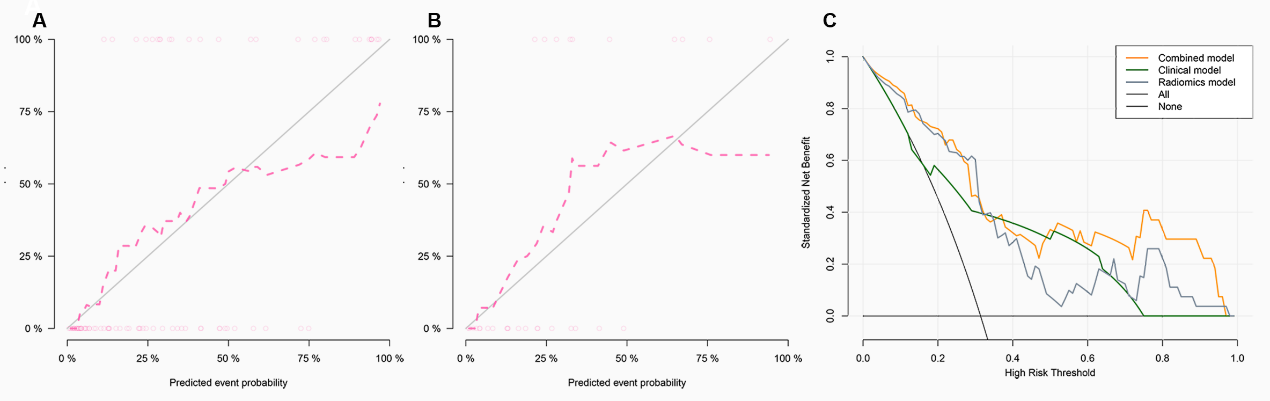
 **Supplementary material 4:** **Figure 1.** Calibrations of the nomogram in the training (A), external validation (B) cohorts. Decision curve analysis for three models (C). The y-axis indicates the net benefit and x-axis indicates threshold probability. The yellow line, grey line, and green line represent net benefit of the radiomics nomogram, the radiomics model and the clinical model, respectively. The radiomics nomogram model had the highest net benefit compared with the other two models.


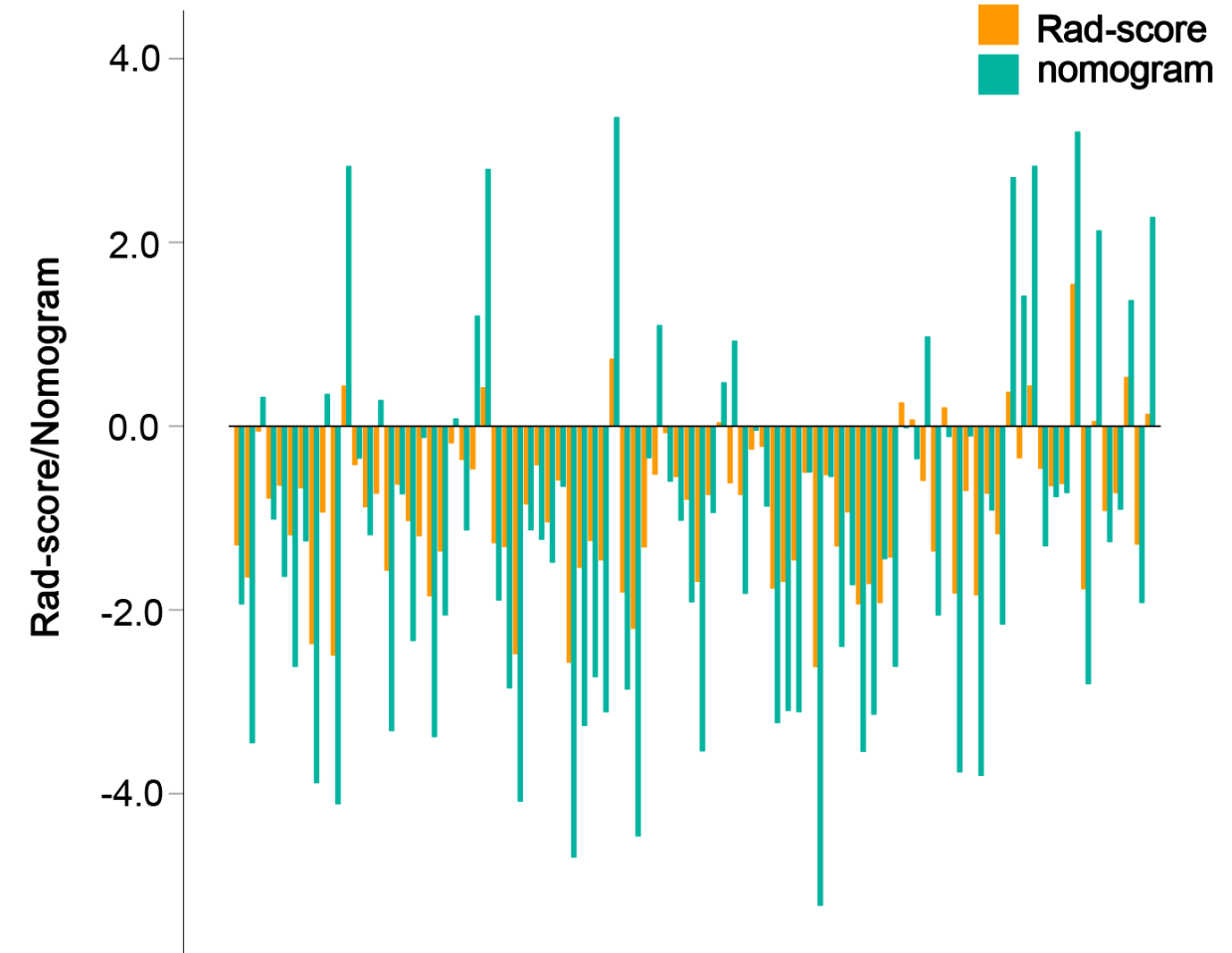


**Supplementary material 5: Figure 2.** The waterfall plot of the rad-score and nomogram points of each patient in training cohort.

**References**

[1] M. Saleh, M. Virarkar, V. Bura, R. Valenzuela, S. Javadi, J. Szklaruk, P. Bhosale, Intrahepatic cholangiocarcinoma: pathogenesis, current staging, and radiological findings, Abdom Radiol (NY) 45(11) (2020) 3662-3680.

[2] H. Wu, Y. Liang, Z. Wang, C. Tan, R. Yang, X. Wei, X. Jiang, Optimizing CT and MRI criteria for differentiating intrahepatic mass-forming cholangiocarcinoma and hepatocellular carcinoma, Acta Radiol (2022) 2841851221113265.

[3] J. Li, Y. Yu, Q. He, The Auxiliary Diagnosis and Imaging Characteristics of MRI Combined with CT in Patients with Cholangiocarcinoma, J Oncol 2021 (2021) 2790958.

[4] K.J. Fowler, A. Sheybani, R.A. Parker, 3rd, S. Doherty, M.B. E, W.C. Chapman, C.O. Menias, Combined hepatocellular and cholangiocarcinoma (biphenotypic) tumors: imaging features and diagnostic accuracy of contrast-enhanced CT and MRI, AJR Am J Roentgenol 201(2) (2013) 332-9.

[5] Y. Kang, J.M. Lee, S.H. Kim, J.K. Han, B.I. Choi, Intrahepatic mass-forming cholangiocarcinoma: enhancement patterns on gadoxetic acid-enhanced MR images, Radiology 264(3) (2012) 751-60.

[6] P. Li, Y. Liang, B. Zeng, G. Yang, C. Zhu, K. Zhao, Z. Xu, G. Wang, C. Han, H. Ye, Z. Liu, Y. Zhu, C. Liang, Preoperative prediction of intra-tumoral tertiary lymphoid structures based on CT in hepatocellular cancer, Eur J Radiol 151 (2022) 110309.
